# Supplementary material for: Exploiting Comparative Omics to Understand the Pathogenic and Virulence-Associated Protease: Anti-Protease Relationships in the Zoonotic Parasites Fasciola hepatica and Fasciola gigantica
Source: Genes (Basel). 2022 Oct 14;13(10):1854. doi: 10.3390/genes13101854 (PMC9601652; doi:10.3390/genes13101854)
Supplement: Supplementary file 1 [file genes-13-01854-s001.zip › Supplementary material.pdf]

Supplementary Materials

Figure S1. Graphical representation of the *F. hepatica* somatic proteome profile relating to peptidase and peptidase inhibitor families. Peptidase and peptidase inhibitor classification is based on MEROPS nomenclature and is detailed in Table S2 and Table S3. Protein proportion is based on PPM abundance extrapolated from the egg data [30] and Exponentially Modified Protein Abundance Index (emPAI) abundance for the remaining stages extrapolated from [27,28]. Life cycle stage abbreviations: Met, metacercariae; NEJ, NEJ 24hr post-excystment; Immature, immature flukes 21 days post infection (dpi). The graphs were generated by ggplot2 in R.

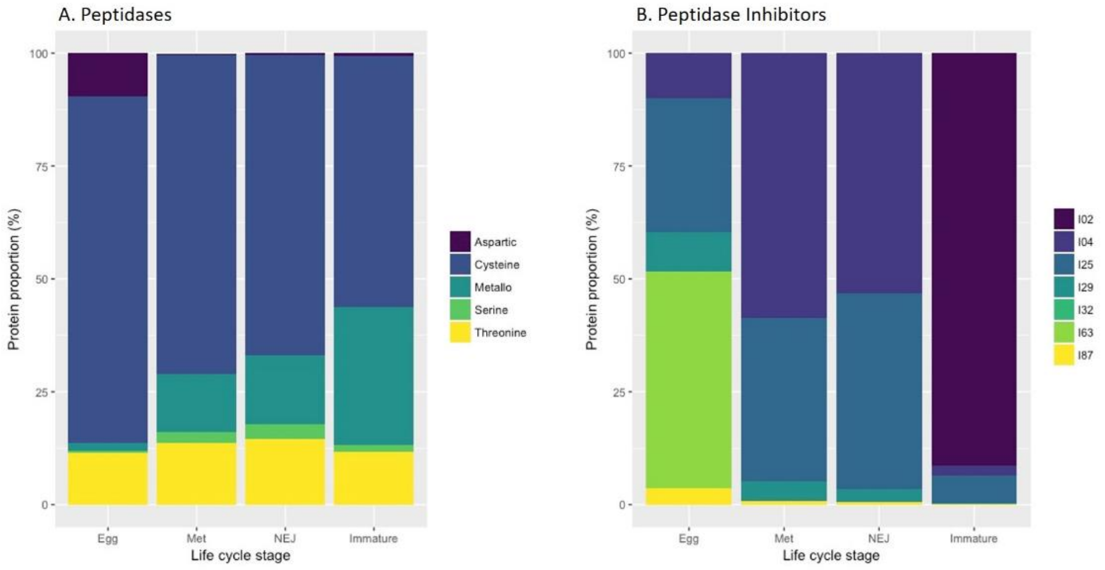

Figure S2. Graphical representation of the *F. hepatica* secretome profile relating to peptidase and peptidase inhibitor families. Peptidase and peptidase inhibitor classification is based on MEROPS nomenclature and is detailed in Table S2 and Table S3. Protein proportion is based on emPAI abundance extrapolated from [27-29]. Life cycle stage abbreviations: NEJ, NEJ 24hr post-excystment; Immature, immature flukes 21 days post infection (dpi). The graphs were generated by ggplot2 in R.

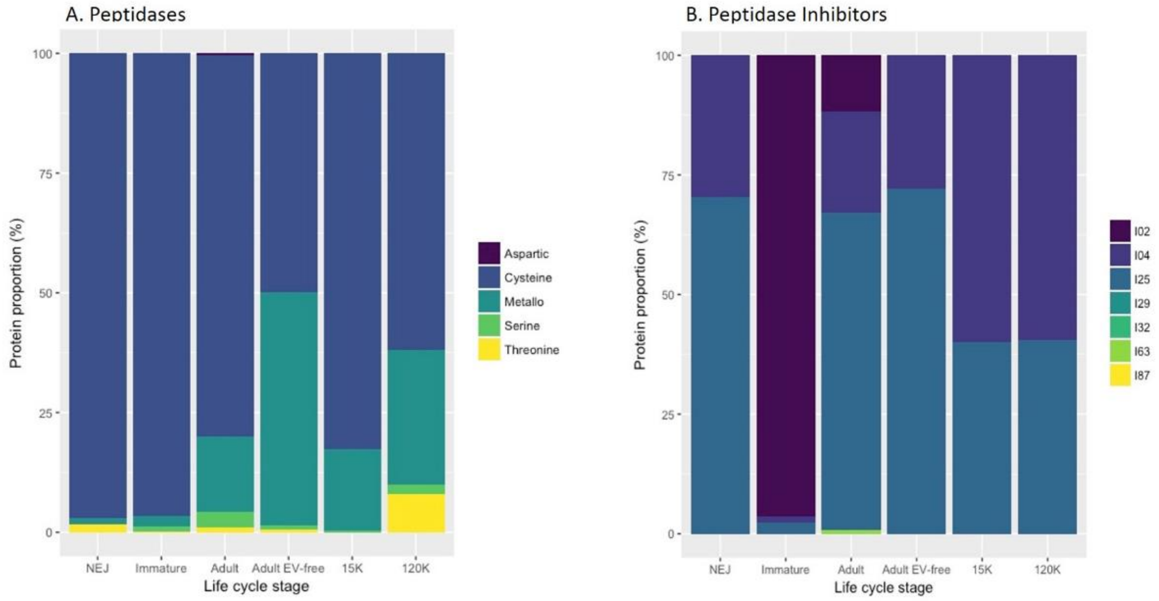

Table S1. Abundant peptidases and peptidase inhibitors secreted by *F. hepatica*

MEROPS id	*F. hepatica* genome accession (PRJEB6687)	*F. hepatica* genome accession (PRJEB25283)	Description	NEJ 24hr	Immature	Adult - EV free	EV_15K	EV_120K
				emPAI values*				
C01A	BN1106_s6570B000050	maker-scaffold10x_1_pilon-snap-gene-0.151	Cathepsin B1 (FhCB1)	1.76	0.63	2.09	0	0.47
C01A	BN1106_s4482B000044	maker-scaffold10x_1_pilon-augustus-gene-0.142	Cathepsin B2 (FhCB2)	1.59	0.52	0	0	0
C01A	BN1106_s6570B000051	maker-scaffold10x_1_pilon-snap-gene-0.151	Cathepsin B3 (FhCB3)	3.58	1.59	7.23	186.01	0
C01A	BN1106_s1840B000150	maker-scaffold10x_889_pilon-snap-gene-0.31	Cathepsin B10 (FhCB10)	0.55	2.25	4.81	0	0.61
C01A	BN1106_s5163B000012	maker-scaffold10x_889_pilon-snap-gene-0.33	Cathepsin B11 (FhCB11)	5.90	2.04	0	0	0
C01A	BN1106_s8462B000006		Cathepsin B-like (FhCB-like_5)	1.08	1.00	1.72	1.63	0.51
C01A	BN1106_s14666B000004		Cathepsin B-like (FhCB-like_2)	2.54	1.85	0	0	1.92
C01A	BN1106_s9304B000006	maker-scaffold10x_1382_pilon-snap-gene-0.52	Cathepsin B4 (FhCB4)	0	8.29	12.89	0	3.40
C01A	BN1106_s7289B000014	maker-scaffold10x_685_pilon-snap-gene-0.26	Cathepsin L1 (FhCL1_2)	0	1.25	14.32	0	2.19
C01A	BN1106_s10332B000011	maker-scaffold10x_2740_pilon-augustus-gene-0.1	Cathepsin L1 (FhCL1_3)	0.18	1.10	17.45	0.65	44.67

C01A	BN1106_s8490B000026	maker-scaffold10x_1194_pilon-snap-gene-0.35	Cathepsin L1 (FhCL1_5)	0.25	5.59	97.53	816.87	44.55
C01A	BN1106_s8098B000020	maker-scaffold10x_819_pilon-augustus-gene-0.18	Cathepsin L2 (FhCL2)	0.30	17.13	55.62	377.07	60.05
C01A	BN1106_s19975B000004	maker-scaffold10x_66_pilon-snap-gene-0.21	Cathepsin L3 (FhCL3_1)	63.71	17.56	0	0	0
C01A	BN1106_s10139B000014	maker-scaffold10x_66_pilon-snap-gene-0.21	Cathepsin L3 (FhCL3_3)	4.22	0.16	18.63	0	0
C01A	BN1106_s4187B000061	maker-scaffold10x_819_pilon-augustus-gene-0.20	Cathepsin L3 (FhCL3_4)	20.60	52.00	1.83	0	0
C01A	BN1106_s3008B000074	maker-scaffold10x_819_pilon-augustus-gene-0.20	Cathepsin L3 (FhCL3_4)	8.13	22.32	1.81	0	0
C01A	BN1106_s6354B000017	maker-scaffold10x_819_pilon-augustus-gene-0.22	Cathepsin L (FhCL_8)	0	1.17	25.08	0.96	2.00
C01A	BN1106_s4636B000039	maker-scaffold10x_147_pilon-snap-gene-0.16	Cathepsin L5 (FhCL5)	0	1.02	16.92	0	0.69
C13	BN1106_s7612B000030	maker-scaffold10x_1606_pilon-snap-gene-0.2	Legumain 1 (FhLeg1)	50.42	8.05	0.67	0	0
C13	BN1106_s2087B000065		Legumain 2 (FhLeg2)	1.81	2.08	1.86	0	0
C13	BN1106_s4223B000091	maker-scaffold10x_375_pilon-snap-gene-0.43	Legumain 3 (FhLeg3)	0.33	4.43	10.48	0.66	0.88
C56	BN1106_s1971B000297	augustus_masked-scaffold10x_313_pilon-processed-gene-0.108	4-methyl-5(B-hydroxyethyl)-thiazole monophosphate	0.51	0.13	26.26	0.52	10.85

			biosynthesis protein					
M17	BN1106_s617B000566 / BN1106_s617B000567	maker-scaffold10x_237_pilon-snap-gene-0.39	Leucine aminopeptidase	2.46	2.22	90.34	190.13	56.84
M17	BN1106_s7079B000034	maker-scaffold10x_75_pilon-snap-gene-0.117	Leucine aminopeptidase	0.58	0.69	15.18	29.55	16.43
M24	BN1106_s468B000343	maker-scaffold10x_2005_pilon-snap-gene-0.39	Xaa-Pro dipeptidase	0	0.12	17.60	22.60	3.36
M49	BN1106_s13034B000002	maker-scaffold10x_296_pilon-snap-gene-0.33	Dipeptidylpeptidase	0.39	0.03	22.38	1.72	0.61
S28	BN1106_s3518B000132	maker-scaffold10x_205_pilon-snap-gene-0.60	Lysosomal pro-X carboxypeptidase	0.15	0.83	11.88	0	3.71
I02	BN1106_s318B000274	maker-scaffold10x_201_pilon-pred_gff_StringTie-gene-0.36	Kunitz-type inhibitor (FhKT1.2)	0	128.65	0	0	0
I02	BN1106_s8826B000029		Kunitz-type inhibitor (FhKT1.1)	0	5.62	0	0	0
I04	BN1106_s3226B000049	maker-scaffold10x_114_pilon-snap-gene-0.90	Serpin 3 (FhSrp3)	1.62	0.13	4.64	5.74	1.23
I25	BN1106_s1612B000138	maker-scaffold10x_327_pilon-snap-gene-0.32	Multi-domain cystatin (FhCys1)	0.48	0.82	37.67	1.79	2.32
I25	BN1106_s4651B000094	maker-scaffold10x_815_pilon-snap-gene-1.92	Stefin 1 (FhStf1)	10.51	2.12	21.87	23.30	10.65
I25	BN1106_s247B000268		Stefin 3 (FhStf3)	0.59	1.19	0.97	0	0

Shaded values represent proteins within top 50 proteins secreted by each specific life cycle stage based on emPAI values.

* Data extrapolated from [27-29].
